# TAPBPR promotes editing of the HLA-B44 peptide repertoire, increasing the presentation of peptides containing a C-terminal tryptophan

**DOI:** 10.3389/fimmu.2025.1685705

**Published:** 2025-10-31

**Authors:** Aure Aflalo, Arwen F. Altenburg, Marcel Wacker, Esam Khanfar, Jens Bauer, Juliane S. Walz, Louise H. Boyle

**Affiliations:** ^1^ Department of Pathology, University of Cambridge, Cambridge, United Kingdom; ^2^ Department of Peptide-based Immunotherapy, Institute of Immunology, University and University Hospital Tübingen, Tübingen, Germany; ^3^ Cluster of Excellence iFIT (EXC2180) “Image-Guided and Functionally Instructed Tumor Therapies”, University of Tübingen, Tübingen, Germany; ^4^ German Cancer Consortium (DKTK), Partner Site Tübingen, A Partnership Between DKFZ and University Hospital, Tübingen, Germany; ^5^ Clinical Collaboration Unit Translational Immunology, Department of Internal Medicine, University Hospital Tübingen, Tübingen, Germany

**Keywords:** MHC, HLA, TAPBPR, antigen presentation, immunopeptidomics

## Abstract

Major histocompatibility complex class I (MHC-I) molecules play a key part in the adaptive immune response by presenting antigens to CD8^+^ T cells. The high degree of polymorphism in MHC-I leads to significant variation in their dependence on the components of the antigen processing and presentation pathway, such as the transporter associated with antigen processing (TAP) and tapasin, and their affinity for the peptide editor TAP-binding protein related (TAPBPR). Here, we investigated the influence of TAPBPR on the cell surface phenotype and peptide repertoire presented by two human leukocyte antigen (HLA) class I allotypes, HLA-B*44:02 and HLA-B*44:05, which are known to differ drastically in their dependence on tapasin. TAPBPR bound weakly to both HLA-B*44:02 and HLA-B*44:05. In contrast to tapasin depletion, the loss of TAPBPR has a limited effect on the cell surface expression of these two molecules. Analysis of the immunopeptidomes presented in the presence and absence of TAPBPR revealed that TAPBPR expression restricted the peptide repertoire presented on HLA-B*44:05, while it diversified the repertoire presented on HLA-B*44:02. Overall, TAPBPR improved the predicted affinity of the peptides displayed on both the HLA-B44 molecules. Furthermore, TAPBPR enhanced the presentation of peptides containing a C-terminal tryptophan residue. Our results show that TAPBPR can significantly impact the peptide repertoire of MHC-I molecules to which it binds weakly. Furthermore, this represents the first study that points to a role for TAPBPR in selecting a specific peptide sequence on MHC class I molecules.

## 1 Introduction

Major histocompatibility complex class I (MHC-I) molecules play a crucial role in the adaptive immune response by presenting antigenic peptides typically derived from the intracellular proteome to CD8^+^ T cells (1, 2). Furthermore, the expression of MHC-I is monitored by natural killer (NK) cells for immunosurveillance (3, 4). The peptides presented on MHC-I are generated and acquired via the antigen processing and presentation (APP) pathway, which involves the proteasome, endoplasmic reticulum (ER) aminopeptidases (ERAPs), the transporter associated with antigen processing (TAP), and the folding chaperones calnexin, calreticulin, and ERp57, together with the peptide editors tapasin and the tapasin-related protein TAPBPR (2, 5).

Peptide editors function by promoting peptide exchange on MHC-I molecules (6, 7). A common feature of both tapasin and TAPBPR is their ability to widen the MHC-I peptide binding groove (8–12). This results in the dissociation of the low-affinity peptide from MHC-I. While being held open in a peptide-receptive conformation by the peptide editors, MHC-I molecules can associate with incoming peptide cargo. If a peptide with high affinity binds to the MHC-I, the resulting conformational change causes the release of the peptide editor. A significant difference in the two MHC-I peptide editors is the environment in which they perform peptide exchange, with tapasin functioning in the peptide-rich environment of the peptide-loading complex (PLC) (13–15) and human TAPBPR functioning outside of this complex, likely in a relatively peptide-deficient environment (5, 16). Tapasin and TAPBPR also differ in the sequence of their peptide editing loops, a key function region of the peptide editors located near the F-pocket of MHC-I (10, 14, 16–18). Together, these functional differences have implications for how the two peptide editors shape the immunopeptidome displayed on MHC-I; tapasin generally plays a key role in the loading of peptides onto MHC-I, while TAPBPR appears to play a more subtle role and refines the peptide repertoire displayed on MHC-I (6, 17, 19).

The exceptionally high level of polymorphism in human MHC-I genes results in a spectrum of their dependence on components of the APP pathway for adequate peptide acquisition and presentation at the cell surface. The dependence of human leukocyte antigen (HLA) class I (HLA-I) allotypes for tapasin has been well-characterized over the years (20–24), and a clear hierarchy of HLA tapasin dependency has been established (21). An intriguing finding from this work is the extreme difference in the tapasin dependency of HLA-B*44:02 and HLA-B*44:05 (23–25). These two allotypes are closely related, differing only at residue 116, which is an aspartate in HLA-B*44:02 and a tyrosine in HLA-B*44:05. Whereas HLA-B*44:02 is highly unstable in its peptide-receptive form (24), leading to a high dependence on tapasin for cell surface expression (21, 23), HLA-B*44:05 has been reported as virtually independent of tapasin, potentially owing to its high conformational stability in a peptide-receptive state (21, 23, 24).

In the case of TAPBPR, a hierarchy of TAPBPR binding to HLA has been established, with HLA-A molecules binding more strongly to TAPBPR than HLA-B and HLA-C molecules (26). However, no drastic impact on MHC-I cell surface expression and stability has, as yet, been demonstrated in cells lacking TAPBPR (5, 6). Therefore, immunopeptidomics, a mass spectrometry-based approach to establish the peptide repertoire, is a key method to assess the impact of TAPBPR on MHC-I (6, 17).

As it is now well established that there are two MHC-I peptide editors, the tapasin dependency hierarchy of MHC-I needs re-evaluation to take TAPBPR as a second editor into account. Here, using HLA-B*44:02 and HLA-B*44:05 as a model system, we aimed to explore whether the tapasin independence of MHC-I molecules is due to their ability to obtain peptide via TAPBPR, while tapasin dependence results from the inability of MHC-I to interact with TAPBPR. To this end, we explored the effect of the loss of TAPBPR on the cell surface expression and the immunopeptidome presented on HeLaM cells modified to express single classical HLA-I genes (26): either HLA-B*44:02 or HLA-B*44:05. This optimized system allows for a more precise evaluation of the selection criteria exerted on peptide: MHC-I by TAPBPR.

## 2 Materials and methods

### 2.1 Cell lines

HeLaM cells with HLA-ABC knocked out (HLA-ABC^KO^) were generated previously, with HLA-ABC loss on the cell surface verified by flow cytometry and total HLA depletion characterized by Western blotting analysis (27). HeLaM HLA-ABC^KO^ cells reconstituted with individual HLA-I alleles (26) and HEK293T cells (a kind gift from Paul Lehner, University of Cambridge) were cultured in Dulbecco’s Modified Eagle’s Medium (Gibco™ DMEM; CAT: 41966052 Thermo Fisher Scientific, Paisley, Renfrewshire, UK) supplemented with 10% fetal bovine serum (FBS; Gibco; CAT: 10500064) and 1% penicillin/streptomycin (Pen/Strep; Gibco; CAT: 15140122) at 37 °C with 5% CO_2_. All cells were regularly tested for the absence of mycoplasma using the MycoAlert kit (Lonza; CAT: LT07-318, Lonza Group AG Basel, Switzerland). Where indicated, cells were stimulated with 200 U/mL human IFNγ (PeproTech, Thermo Fisher UK Limited, Altrincham, Cheshire, UK; CAT: 300-02) for 48–72 h before an experiment to upregulate components of the APP pathway.

### 2.2 Depletion of TAPBPR using CRISPR–Cas9 transfection

TAPBPR knock-down (TAPBPR^KD^) cell lines were generated as previously described (6, 28). Briefly, 10^5^ HeLaM cells/well were seeded in a 6-well plate 24 h before transfection. Cells were transfected with 3 µg pSpCas9(BB)-2A-Puro plasmid containing one of two sgRNAs: CRISPR7 (GCGAAGGACGGTGCGCACCG), which targets exon 2 of the *TAPBPL* gene, or CRISPR9 (CGATTTCCAAGGGGGCACAC), which targets exon 3. Transfection was performed using the FuGENE HD transfection reagent (Promega UK Ltd., Chilworth, Hampshire, Uk; CAT: E2311). After 24 h, the culture medium was replaced with fresh media containing 2 µg/mL puromycin. Puromycin selection was maintained for 48 h.

### 2.3 Depletion of tapasin using CRISPR–Cas9 RNP electroporation

The crRNA Hs.Cas9.TAPBPR.1.AA (AACCAACACTCGATCACCGC) targeting exon 1 of the *TAPBP* gene was obtained from Integrated DNA Technologies UK Ltd., London, UK. crRNA and tracrRNA labelled with the ATTO-550 fluorophore (Integrated DNA Technologies; CAT: 1075928) were mixed at a 1:1 ratio and incubated at 95 °C for 5 min to produce guide RNA. Ribonucleoprotein (RNP) complexes were produced by combining 0.48 nmol guide RNA with 34 µg recombinant *Streptococcus pyogenes* Cas9 protein (Integrated DNA Technologies; CAT: 1081059) in a total volume of 10 µL RNP complex in phosphate-buffered saline (PBS). A total of 0.8 × 10^6^ cells from a confluent culture were washed to remove FBS and resuspended in 80 µL Nucleofector+ solution (Lonza Kit R; CAT: VVCA-1001), containing Nucleofector supplement at a 1:4.5 ratio. The RNP complex and 1 µL electroporation enhancer (Integrated DNA Technologies; CAT: 1075916) were added to the cells, and the cell suspension was transferred to an electroporation cuvette. Cells were electroporated using program I-013 on the Nucleofector 2b device (Lonza). Immediately after electroporation, 500 µL pre-warmed culture media was added to the cells, and the cells were transferred to a 6-well plate containing pre-warmed media. After 24 h, cells were expanded, and electroporation efficiency was assessed using flow cytometry by measuring ATTO-550 fluorescence. Cells were cultured for 7–10 days before phenotyping.

### 2.4 Lentiviral transduction to overexpress TAPBPR in HeLa cells

Lentiviral transduction was used to overexpress human TAPBPR in HeLaM-HLA-B*44:02 and HeLaM-HLA-B*44:05 cells as previously described (5, 26). To produce lentivirus, HEK293T cells were seeded in 6-well plates at least 24 h before transfection. For each transfection, 0.8 mg pHRSIN-C56W-UbEM vector containing the full-length human TAPBPR sequence (5), and 0.5 µg each of the packaging vector pCMVΔR8.91 and the envelope vector pMD.G were used, together with 4.5 µL of the FuGENE HD (Promega; CAT: E2311) transfection reagent. Target cells were seeded in 6-well plates at least 24 h before transduction. At 48 h and 72 h post-transfection, supernatant containing lentivirus was harvested from the HEK293T cells, centrifuged, and filtered to remove cell debris. The supernatant volume was adjusted to 2 mL with fresh complete DMEM, and 8 μg/mL polybrene (Sigma Aldrich, Merck Life Science UK Ltd., Gillingham, Dorset) was added before addition to the target cells for transduction.

### 2.5 Antibodies

PeTe4, a mouse monoclonal antibody (mAb) raised against the luminal domain of TAPBPR (5), was used to immunoprecipitate TAPBPR. For Western blotting, OTI1C9 antibody against TAPBPR (Abcam Ltd., Cambridge, Cambridgeshire, UK), a polyclonal antibody against MHC-I (Proteintech; CAT: 15240-1-AP), and a polyclonal antibody against calnexin (Enzo Life Sciences UK Ltd., Exeter, Devon, UK; CAT: ADI-SPA-860) were used as primary antibodies, followed by secondary antibodies recognizing mouse and rabbit IgG conjugated to LICOR IRDye 800 CW or LICOR IRDye 680 RD (LI-COR Biosciences UK Ltd., Cambridge, Cambridgeshire, UK; CAT: 926–32210 and 926-68071). For flow cytometry, the following antibodies were used: W6/32 (Biolegend UK Ltd., London, UK; CAT: 311417), a polyclonal antibody against MHC-I (Proteintech Europe, Manchester, UK; CAT: 15240-1-AP); 4E, mouse mAb, which detects HLA-B molecules (29) (a kind gift from Peter Cresswell, Yale University); PeTe6, a mouse mAb raised against the luminal domain of TAPBPR (5); and PasTa1, a mouse mAb raised against tapasin (a kind gift from Peter Cresswell, Yale University). PeTe6 and PasTa1 were conjugated in-house to Alexa Fluor 680 (Thermo Fisher; CAT: A20188) and Alexa Fluor 594 (Thermo Fisher UK Limited, Altrincham, Cheshire, UK; CAT: A20185), respectively, according to the manufacturer’s instructions. A secondary antibody against rabbit IgG conjugated to Pacific Orange (Invitrogen™, Thermo Fisher UK Limited, Altrincham, Cheshire, UK; CAT: P31584) was used to detect the polyclonal anti-MHC-I antibody.

### 2.6 TAPBPR immunoprecipitation, gel electrophoresis, and Western blotting

Cells were washed with PBS, and Triton lysis buffer [1% Triton X-100 (VWR International Ltd., Lutterworth, Leicestershire, UK) in Tris-buffered saline (TBS) (20 mM Tris-HCl, 150 mM NaCl, 2.5 mM CaCl_2_) supplemented with protease inhibitor cocktail (Roche Diagnostics Ltd., Burgess Hill, Sussex, UK CAT: 04693159001)] was added directly to the culture plate. Cells were removed from the plate by scraping, and lysis was allowed to proceed for 30 min at 4 °C, with rotation. Lysates were centrifuged for 20 min at 11,000 *g* at 4 °C to remove cell debris, and supernatant was collected. TAPBPR was pulled down using 5 µg PeTe4 conjugated to 50 µL Protein A Sepharose beads per sample for 90 min at 4 °C, with rotation. Beads were washed four times with 0.1% Triton lysis buffer diluted in TBS to remove unbound protein, then denatured and reduced in sample loading buffer (25 mM Tris-HCl, pH 6.8, 4% SDS Sodium dodecyl sulfate, 20% glycerol, and 0.04% bromophenol blue), and supplemented with 100 mM β-mercaptoethanol, at 99 °C for 10 min.

Following separation by Sodium Dodecyl Sulfate-Polyacrylamide Gel Electrophoresis (SDS–PAGE), proteins were transferred onto a nitrocellulose membrane (GE Healthcare; Global Life Sciences Solutions Operations Ltd., Little Chalfont, Buckinghamshire, UK CAT: 10600004) using a Trans-Blot Turbo semi-dry transfer system (Bo-Rad Laboratories Ltd., Watford, Hertfordshire, UK). Membranes were blocked for 30 min in 5% (w/v) dried milk in PBST [PBS supplemented with 0.1% Tween 20 (Sigma Aldrich; CAT: P1379)]. Primary antibody staining was performed for 1 h at room temperature or overnight at 4 °C, with rotation. After washing with PBST, membranes were stained with secondary antibody in 5% milk in PBST for 1 h at room temperature. After washing with PBST, fluorescence was detected using a LI-COR Odyssey (LI-COR Biosciences UK Ltd., Cambridge, Cambridgeshire, UK) or Typhoon (Cytiva Global Life Sciences Solutions Operations Ltd., Little Chalfont, Buckinghamshire, UK) imager and analyzed using the ImageJ software.

### 2.7 Flow cytometry

For analysis by flow cytometry, cells were detached from the culture vessel using trypsin-EDTA solution (Gibco; CAT: 25300054) and washed in fluorescence-activated cell sorting (FACS) buffer [PBS containing 2% FBS and 5 mM ethylenediaminetetraacetic acid (EDTA)]. For extracellular staining, cells were incubated for 30 min at 4 °C with the primary antibody diluted in FACS buffer. Cells were washed once with FACS buffer to remove excess antibody and then incubated with secondary antibody diluted in FACS buffer for a further 30 min at 4 °C, where necessary.

For intracellular staining, cells were washed once with FACS buffer to remove media. When no extracellular staining was included in the protocol, cells were fixed and permeabilized using the BD Cytofix/Cytoperm kit (BD Biosciences; Becton Dickinson UK Ltd., Wokingham, Berkshire CAT: 10482735) according to the manufacturer’s instructions. Antibody staining was then performed for 30 min at 4 °C with antibodies diluted in BD Perm/Wash solution from the same kit. After staining, cells were washed once in BD Perm/Wash solution and once in FACS buffer. Fluorescence was detected using a Cytoflex S flow cytometer (Beckman Coulter UK Ltd., Little Chalfont, Buckinghamshire, UK), and data were analyzed using the FlowJo software (v10) (FlowJo).

### 2.8 MHC-I immunopeptidomic analysis

A total of 5 × 10^8^ cells were grown for each sample, within six passages of characterizing TAPBPR expression in the cells. Cells were stimulated with 200 U/mL IFNγ for 48–72 h before harvesting. Cells were harvested using trypsin-EDTA, washed twice in PBS, pelleted, and snap-frozen. Pellets were kept at −70 °C before peptide isolation.

#### 2.8.1 Isolation of MHC-I peptides

HLA immunopurification was performed via immunoaffinity chromatography using the pan-HLA class I-specific mAb W6/32 (produced in-house) as previously described (30). A 3-{dimethyl[3-(3α,7α,12α-trihydroxy-5β-cholan-24-amido)propyl]azaniumyl}propane-1-sulfonate (CHAPS)-based lysis buffer [1.2% (w/v) in PBS, supplemented with protease inhibitor cocktail containing EDTA (Roche)] was used, while the immunoprecipitation was conducted in Econo-Column Chromatography Columns (0.5 cm × 5 cm; Bio-Rad), followed by acidic elution with 0.2% (v/v) trifluoroacetic acid (TFA), size-exclusion filtration with an Amicon Ultra 0.5 centrifugal filter unit (Merck Millipore, Merck Life Science UK Ltd., Gillingham, Dorset, UK), and a subsequent desalting step using a ZipTip C_18_ pipette tip (Merck Millipore).

#### 2.8.2 Analysis of MHC-I peptides by mass spectrometry

Isolated peptides were analyzed in five technical replicates. Reversed-phase liquid chromatography (nanoUHPLC, UltiMate 3000 RSLCnano, Thermo Fisher Scientific) was performed applying a gradient ranging from 2.4% to 32.0% of acetonitrile (ACN) over 90 min for sample separation coupled online to an Orbitrap Fusion Lumos mass spectrometer (Thermo Fisher Scientific) using data-dependent acquisition and a collision-induced dissociation method generating fragment spectra with a mass range limited to 400–650 m/z and positive charge states 2–3.

#### 2.8.3 Database search and peptide filtering

Spectra were annotated by database search of the human proteome (SwissProt database, v130927) using the Sequest HT search engine, allowing oxidized methionine as a dynamic modification. Peptides were filtered to allow a length of 8–12 amino acids, and the false discovery rate was set to 5%. HLA binders were identified for each allotype as having either a percentile rank of ≤2% using NetMHCpan 4.1 (31) or a score ≥60% using the SYFPEITHI database (32). Further analysis of peptidomes was performed using an in-house Python script using the NumPy, pandas, matplotlib, seaborn, and Logomaker packages (33–37).

#### 2.8.4 Label-free quantitation

Label-free quantitation was performed as previously described (17). Briefly, the peptide amount for all samples was normalized by cell count before mass spectrometry analysis. For each pair of conditions analyzed, peptide lists from the five replicates of both conditions were merged and filtered for HLA binders as above. Peptide-spectrum matches (PSMs), corresponding scores, and intensities (area under the curve of the extracted ion chromatogram of precursor ions) for each replicate were extracted from the unfiltered data and included in the peptide list. For each peptide and each condition, the mean area under the curve across all Label-Free Quantification Mass Spectrometry (LFQ-MS) runs was calculated and used to determine the fold change of mean area under the curve in condition A versus condition B. Peptides not detected in at least two technical replicates were excluded from Volcano plots. The fold change of peptides detected only in one condition was calculated by replacing zero values with the median of the five least intensities, representing the limit of detection specific to each sample. Further, a normalization step computing PSM intensities in proportion to the total intensity of precursor ions in technical replicates was included. Statistical significance was calculated using a two-tailed t-test with correction for multiple comparisons using the Benjamini–Hochberg method. The log2(fold change) and adjusted p-values were visualized as volcano plots using an in-house Python script using the NumPy, pandas, matplotlib, and seaborn packages (33–36). Significant enrichment was set at an adjusted p-value of <0.01 for any up- or downmodulated peptides [log2(fold change) >2 or <−2].

### 2.9 MHC-I binding peptides

Based on the peptide EEFGRAFSF known to bind to both HLA-B*44:02 and HLA-B*44:05 (36, 37), the following peptides were purchased from Peptide Synthetics (Peptide Protein Research Ltd., Southampton, Hampshire, UK), UK: EEFGK*AFSF, EEFGK*AFSL, and EEFGK*AFSW. K* indicates a lysine labelled with 5-carboxytetramethylrhodamine (TAMRA).

### 2.10 Peptide loading assay with overexpressed cell surface TAPBPR

A total of 25,000–35,000 HeLaM variant cells (as indicated in the figure legend) were seeded in 12-well plates 48–72 h before the experiment and stimulated with 200 U/mL IFNγ, where indicated. Cells were washed once with PBS and then incubated with fluorescent peptide in 300 µL Opti-MEM (Gibco) for 1 h at 37 °C. Cells were then washed three times with PBS to remove unbound peptide, harvested, and stained for cell surface TAPBPR as above. Samples were analyzed using a Cytoflex S flow cytometer (Beckman Coulter), and data were analyzed using the FlowJo software (v10) (FlowJo).

## 3 Results

### 3.1 HLA-B*44:02 and HLA-B*44:05 bind very weakly to TAPBPR

**Figure 1:**
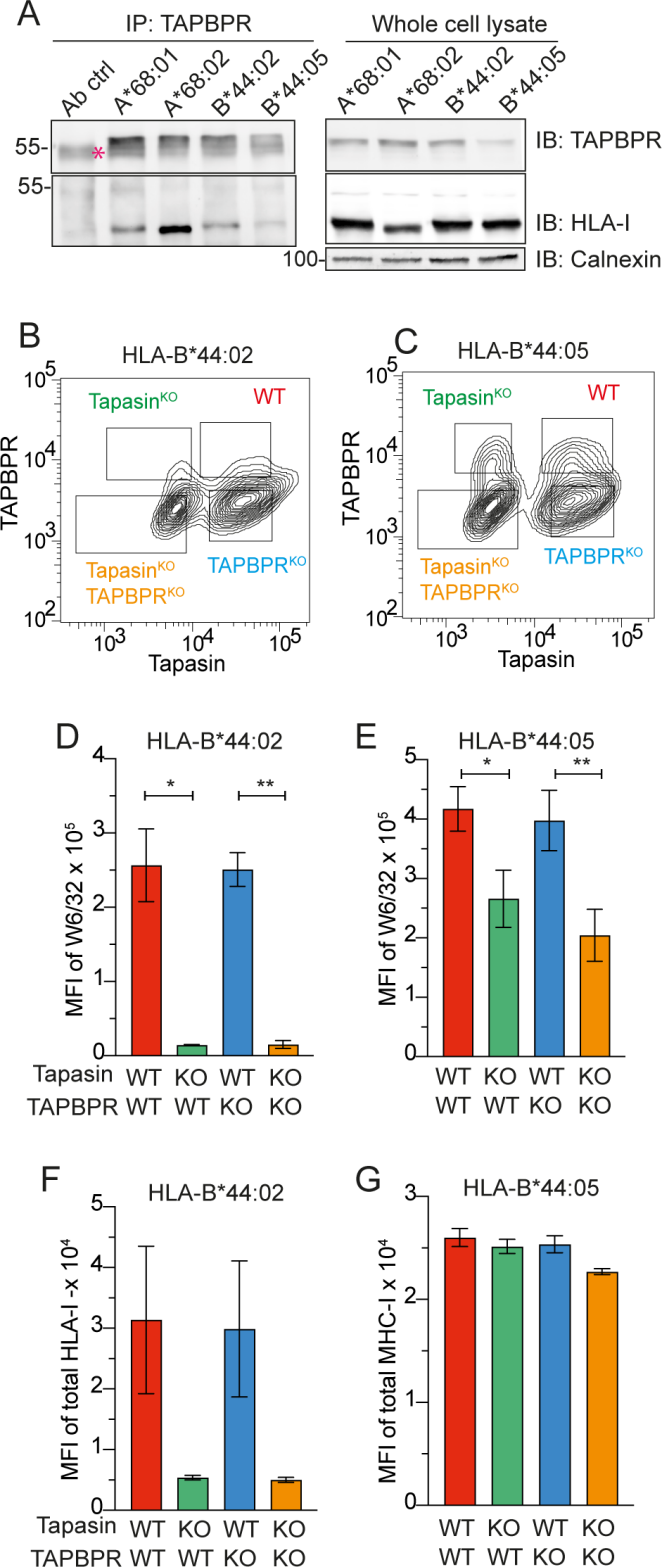
Effect of loss of tapasin and TAPBPR on cell surface expression of HLA-B*44:02 and HLA-B*44:05. **(A)** Both HLA-B*44:02 and HLA-B*44:05 co-immunoprecipitate with TAPBPR. TAPBPR was immunoprecipitated using PeTe4 in IFN-γ-treated HeLaM HLA-ABC^KO^ cells expressing different MHC-I allotypes, and MHC-I association was assessed using Western blotting. Calnexin was used as a loading control, and a sample containing only antibody and beads (Ab control) was used as a negative control. Red asterisk indicates the location of the PeTe4 antibody heavy band used in the immunoprecipitation. Data are representative of two independent experiments. **(B, C)** Gating strategy to identify tapasin^WT^:TAPBPR^WT^, tapasin^KO^, TAPBPR^KO^, and tapasin^KO^:TAPBPR^KO^ subpopulations in HeLa HLA-ABC^KO^ tapasin^KD^:TAPBPR^KD^ cells expressing **(B)** HLA-B*44:02 or **(C)** HLA-B*44:05. **(D**–**G**) Effect of loss of tapasin and/or TAPBPR on cell surface expression of **(D, F)** HLA-B*44:02 and **(E, G)** HLA-B*44:05. Cell surface HLA-B*44:02 and HLA-B*44:05 levels were measured in each of the four subpopulation by staining with **(D, E)** W6/32, which recognizes the conformational forms of HLA associated with peptide, or **(F, G)** with a polyclonal antibody against MHC-I, which recognizes MHC-I in any conformation. In all experiments, cells were pre-stimulated with IFNγ. Bars show MFI ± SD from two independent experiments. * p < 0.05 and ** p < 0.01 using a one-way ANOVA followed by unpaired Welch’s t-test. IB, immunoblot; HLA, human leukocyte antigen; KO, knockout; MFI, mean fluorescence intensity; TAPBPR, transporter associated with antigen processing; WT, wild type; MHC-I, major histocompatibility complex class I.

To investigate the interaction between TAPBPR and HLA-B*44:02 and HLA-B*44:05, immunoprecipitation experiments were performed on HLA-ABC knockout (HLA-ABC^KO^) HeLaM cells (27) reconstituted with individual HLA-I alleles (26). Upon IFNγ treatment, the expression of HLA-B*4402 and HLA-B*44:05 was similar to that of the naturally expressed HLA-B allotype in HeLaM cells (**Supplementary Figure 1A, B**). In contrast to the strong interaction observed with HLA-A*68:02 and also HLA-A*68:01 (9), very little HLA-B*44:02 and HLA-B*44:05 was associated with TAPBPR (**Figure 1A**). These results indicated that TAPBPR binds very weakly or transiently to both HLA-B*44:02 and HLA-B*44:05.

### 3.2 Neither HLA-B*44:02 nor HLA-B*44:05 is dependent on TAPBPR for surface expression

To investigate if TAPBPR played any role in antigen presentation on HLA-B*44:02 and HLA-B*44:05, we generated TAPBPR knock-down (TAPBPR^KD^) versions of the HeLaM HLA-ABC^KO^ cells expressing HLA-B*44:02 or HLA-B*44:05. As HLA-B*44:02 and HLA-B*44:05 exhibit distinct tapasin dependence profiles, we also generated cell lines in which both tapasin and TAPBPR were knocked down (tapasin^KD^:TAPBPR^KD^). The knock-down efficiency for tapasin was between 50% and 60% in the various cell lines, with two distinct populations apparent: one with wild-type (WT) tapasin expression and one with tapasin knocked down (**Supplementary Figure 1C, D**). The TAPBPR knock-down was more efficient, with most of the cells targeted depleted of TAPBPR expression, although there was still some residual detection of the protein (**Supplementary Figure 1E, F**). In contrast to the WT cells, which displayed a single population positive for both tapasin and TAPBPR (**Supplementary Figure 1G, H**), the tapasin^KD^:TAPBPR^KD^ cell lines exhibited four populations (**Figures 1B, C**). These comprised cells expressing both tapasin and TAPBPR (WT), cells lacking tapasin only (tapasin^KO^), cells lacking TAPBPR only (TAPBPR^KO^), and cells in which both peptide editors were knocked out (tapasin^KO^:TAPBPR^KO^) (**Figures 1B, C**). We assessed the effect of the loss of tapasin and/or TAPBPR on HLA-B*44:02 and HLA-B*44:05 cell surface expression and stability by analyzing surface HLA-I staining with either W6/32 or a polyclonal antibody raised against HLA-I on these four gated populations (**Figures 1D, G**). As expected, the loss of tapasin resulted in a >10-fold reduction in the cell surface of HLA-B*44:02, as detected with W6/32, which recognizes MHC-I complexed with β2m and peptide (**Figure 1D**, green bar). In the case of HLA-B*44:05, the level of W6/32-reactive peptide-MHC (pMHC) complexes was reduced by approximately 40% in the absence of tapasin (**Figure 1E**, green bar). Thus, the tapasin dependency of HLA-B*44:02 and the relative tapasin independence of HLA-B*44:05 were confirmed in HeLaM cells, as has previously been observed in other cell types/systems (21, 24). In contrast, the loss of TAPBPR had no significant effect on the surface expression of W6/32-reactive HLA-B*44:02 (**Figure 1D**, blue bars) or HLA-B*44:05 levels (**Figure 1E**, blue bars) compared to WT cells. Furthermore, tapasin^KO^:TAPBPR^KO^ cells did not have reduced cell surface expression of HLA-B*44:02 compared to the tapasin^KO^ cells (**Figure 1D**, orange bars). A slight but not statistically significant reduction in HLA-B*44:05 levels in the tapasin^KO^:TAPBPR^KO^ cells compared to the tapasin^KO^ cells was observed (**Figure 1E**, orange bars). These results suggest that TAPBPR and tapasin may play redundant roles in promoting the expression of HLA-B*44:05 at the cell surface.

Interestingly, when staining for total HLA-I levels on the plasma membrane using a polyclonal antibody raised against HLA-I, the loss of tapasin resulted in a >10-fold reduction in the expression of cell surface HLA-B*44:02 (**Figure 1F**, green bar), mirroring the results observed with W6/32. However, the total HLA-B*44:05 surface levels detected with the polyclonal antibody were unchanged in the absence of tapasin (**Figure 1G**, green bar). This indicates the potential presence of non-W6/32 reactive forms of HLA-B*44:05, such as peptide-receptive MHC-I or free heavy chain forms in tapasin-depleted cells.

### 3.3 TAPBPR expression restricts the peptide repertoire presented on HLA-B*44:05

**Figure 2:**
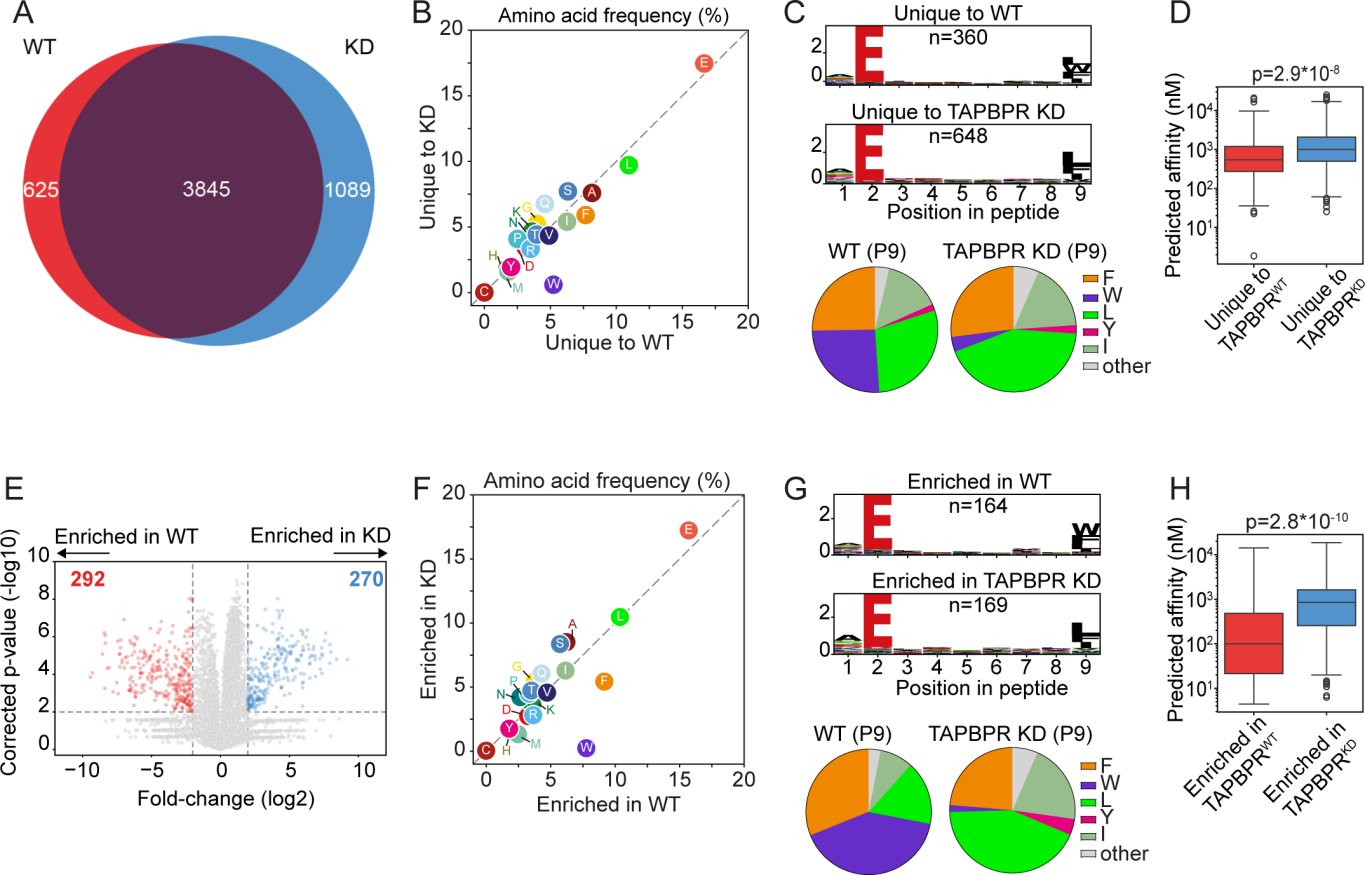
HLA-B*44:05 ligandomes from TAPBPR^WT^ and TAPBPR^KD^ cells. HLA-presented peptides were isolated from W6/32 reactive complexes from IFN-γ-treated HeLaM HLA-ABC^KO^ cells expressing HLA-B*44:05, with either endogenous TAPBPR (TAPBPR^WT^) or TAPBPR-depleted (TAPBPR^KD^). **(A)** Venn diagrams show an overlap analysis of the HLA-presented peptides isolated. **(B)** Frequency (%) of each amino acid from the HLA-presented peptides unique to TAPBPR^WT^ and TAPBPR^KD^ cells. **(C)** SeqLogos showing the consensus sequences of 9mer peptides unique to TAPBPR^WT^ and TAPBPR^KD^ cells, with pie charts displaying the frequency of the amino acids found at the C-terminal residue. **(D)** Boxplots showing the NetMHC-predicted affinities of the HLA-presented peptides unique to TAPBPR^WT^ and TAPBPR^KD^ cells. Statistics obtained using unpaired two-tailed t-tests. **(E)** Volcano plots showing HLA-presented peptide enrichment in TAPBPR^WT^ and TAPBPR^KD^ cells. Red and blue dots indicate peptides statistically significantly enriched in TAPBPR^WT^ and TAPBPR^KD^ cells, respectively. Statistical significance was calculated using a two-tailed t-test with correction for multiple comparisons using the Benjamini–Hochberg method. **(F)** Frequency (%) of each amino acid from peptides enriched in TAPBPR^WT^ and TAPBPR^KD^ cells. **(G)** SeqLogos showing the consensus sequences of 9mer peptides enriched in TAPBPR^WT^ and TAPBPR^KD^ expressing cells, with pie charts displaying the frequency of the amino acids found at the C-terminal residue. **(H)** Boxplots showing the NetMHC-predicted affinities of the peptides enriched in TAPBPR^WT^ and TAPBPR^KD^. Statistics obtained using unpaired two-tailed t-tests. HLA, human leukocyte antigen; KO, knockout; TAPBPR, transporter associated with antigen processing; WT, wild type.

Next, we explored whether TAPBPR played a role in shaping the peptide repertoire presented on the tapasin-independent HLA-B*44:05 by comparing its immunopeptidome from TAPBPR^WT^ and TAPBPR^KD^ cells. There was a large overlap (69%; 3,845/5,559 peptides) between the peptidomes presented on TAPBPR^WT^ and TAPBPR^KD^ cells, which likely represents peptides not influenced by TAPBPR expression for presentation (**Figure 2A**) (**Supplementary Table 1**). The overall number of peptides varied between TAPBPR^WT^ and TAPBPR^KD^, with 1,089 peptides unique to TAPBPR^KD^ cells (i.e., not detected in TAPBPR^WT^ cells) and 625 peptides unique to TAPBPR^WT^ cells (i.e., not detected in TAPBPR^KD^ cells) (**Figure 2A**). There was no noticeable difference in the length of peptides presented on HLA-B*44:05 between peptides uniquely found in the TAPBPR^WT^ and TAPBPR^KD^ cells (**Supplementary Figure 2A**). As a broader immunopeptidome was presented in cells lacking TAPBPR, the presence of TAPBPR appears to restrict the peptide repertoire presented on HLA-B*44:05, as has been observed previously for other HLA-I allotypes (6, 17).

### 3.4 TAPBPR promotes the loading of peptides containing a C-terminal tryptophan residue and improves the affinity of peptides presented on HLA-B*44:05

When comparing the sequence of the peptides presented on HLA-B*44:05 that were unique to TAPBPR^WT^ and TAPBPR^KD^ cells, a noticeable increase in the frequency of tryptophan (W) was observed in peptides unique to TAPBPR^WT^ cells (**Figure 2B**). This tryptophan residue was predominantly located at the C-terminus of peptides presented only by TAPBPR^WT^ cells (**Figure 2C**). TAPBPR promotion of the presentation of peptides containing a C-terminal tryptophan on HLA-B*44:05 was also observed in the 10mer and 11mer peptides (**Supplementary Figure 2B, C**) as well as the 9mer peptides (**Figure 2C**). Interestingly, peptides unique to TAPBPR^WT^ cells exhibited a higher predicted affinity (median: 541 nM) than those unique in TAPBPR^KD^ cells (median: 996 nM) (**Figure 2D**), suggesting that TAPBPR optimizes the peptide repertoire presented on HLA-B*44:05.

As TAPBPR may not only impact the presence/absence of peptides, we also assessed TAPBPR-mediated changes in peptide abundance. Label-free quantitation identified 292 peptides that were enriched in the TAPBPR^WT^ cells and 270 peptides enriched in the TAPBPR^KD^ cells (**Figure 2E**). Enrichment was defined as a minimum four-fold change in mean intensity (area under the curve) between the TAPBPR^WT^ and TAPBPR^KD^ samples and an adjusted p-value of 0.01 (**Figure 2E**, dotted lines). Further analysis on these enriched peptides confirmed that TAPBPR presence promoted the presentation of peptides containing a C-terminal tryptophan (**Figures 2F, G**) (**Supplementary Figure 3B, C** for 10mer and 11mer peptides). A similar improvement in the predicted affinity of peptides presented on HLA-B*44:05 was also observed in the peptides enriched in the TAPBPR^WT^ (median: 100 nM) compared to those enriched in the TAPBPR^KD^ cells (median: 846 nM) (**Figure 2H**). This indicates that TAPBPR promotes the presentation of high-affinity peptides on HLA-B*44:05 molecules.

### 3.5 TAPBPR expression increases the diversity of peptides presented on HLA-B*44:02

**Figure 3:**
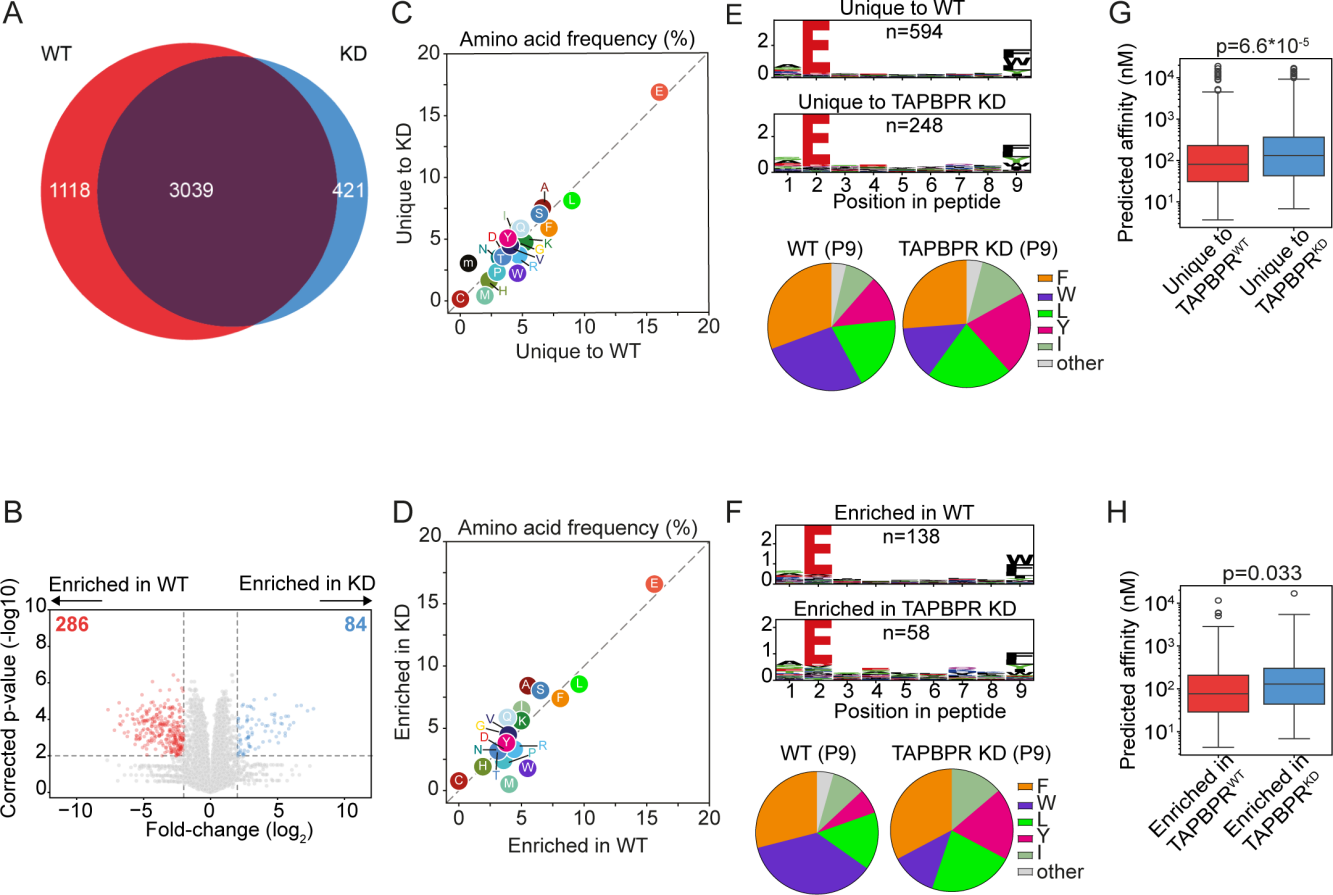
HLA-B*44:02 ligandomes from TAPBPR^WT^ and TAPBPR^KD^ cells. HLA-presented peptides were eluted from W6/32 reactive complexes from IFN-γ-treated HeLa-M HLA-ABC^KO^ cells expressing HLA-B*44:02, with either endogenous TAPBPR (TAPBPR^WT^) or TAPBPR-depleted (TAPBPR^KD^). **(A)** Venn diagrams show the size and overlap of the HLA-presented peptides eluted. **(B)** Volcano plots showing HLA-presented peptide enrichment in TAPBPR^WT^ and TAPBPR^KD^ cells. Red and blue dots indicate peptides significantly enriched in TAPBPR^WT^ and TAPBPR^KD^ cells, respectively. Statistical significance was calculated using a two-tailed t-test with correction for multiple comparisons using the Benjamini–Hochberg method. **(C, D)** Frequency (%) of each amino acid from the HLA-presented peptides **(C)** unique to TAPBPR^WT^ and TAPBPR^KD^ cells or **(D)** enriched in TAPBPR^WT^ and TAPBPR^KD^ cells. **(E, F)** SeqLogos showing the consensus sequences of 9mer peptides **(E)** unique to TAPBPR^WT^ and TAPBPR^KD^ cells or **(F)** enriched in TAPBPR^WT^ and TAPBPR^KD^ expressing cells, with pie charts displaying the frequency of the amino acids found at the C-terminal residue. **(G, H)** Boxplots showing the NetMHC-predicted affinities of the peptides **(G)** unique to TAPBPR^WT^ and TAPBPR^KD^ cells or **(H)** enriched in TAPBPR^WT^ and TAPBPR^KD^. Statistics obtained using unpaired two-tailed t-tests. HLA, human leukocyte antigen; KO, knockout; TAPBPR, transporter associated with antigen processing; WT, wild type.

When we examined how TAPBPR shapes the peptide repertoire presented on HLA-B*44:02, interestingly, we observed a larger immunopeptidome in the presence of TAPBPR, with 1,118 peptides unique to TAPBPR^WT^ cells and only 421 peptides uniquely presented in TAPBPR^KD^ cells (**Figure 3A**) (**Supplementary Table 1**). As for HLA-B*44:05, there was no noticeable difference in the length of peptides presented on HLA-B*44:02 between peptides uniquely found in the TAPBPR^WT^ and TAPBPR^KD^ cells (**Supplementary Figure 2D**). Analyses of the label-free quantitation data, comparing the abundance of peptides between TAPBPR^WT^ and TAPBPR^KD^ that were significantly enriched in either cell line, mirrored this overall enlargement of the HLA-B*44:02 immunopeptidome in TAPBPR^WT^ cells (**Figure 3B**). In HLA-B*44:02 expressing cells, 286 peptides were significantly more abundant in TAPBPR^WT^ cells, compared to only 84 peptides enriched in TAPBPR^KD^ cells (**Figure 3B**). Thus, the loss of TAPBPR leads to a reduction in the number of unique peptides presented by HLA-B*44:02 and the abundance of a subset of peptides. Therefore, the presence of TAPBPR enlarges the repertoire presented by HLA-B* 44:02, indicating it may play an important role in peptide acquisition for HLA-B* 44:02.

### 3.6 TAPBPR improves the affinity of peptides presented on HLA-B*44:02

The comparison of the sequence of peptides presented on HLA-B*44:02 revealed that there was a slight increase in the frequency of tryptophan in the peptides unique to the TAPBPR^WT^ cells compared to the TAPBPR^KD^ cells (**Figure 3C**), as similarly observed with the enriched peptide dataset (**Figure 3D**). However, this was not as striking as observed for HLA-B*44:05 (**Figure 2B**). While the tryptophan residue at the C-terminus of the peptide was presented on HLA-B*44:02 in the absence of TAPBPR, its presentation was enhanced when TAPBPR was present in both the unique (**Figure 3E**, **Supplementary Figure 2E,F**) and enriched peptide datasets (**Figure 3F**, **Supplementary Figure 3E**). These findings suggest that HLA-B*44:02 can obtain peptides containing a C-terminal tryptophan residue in a TAPBPR-independent manner, but TAPBPR presence further promotes the presentation of such peptides.

Peptides uniquely presented on HLA-B*44:02 in TAPBPR^WT^ cells exhibited a higher predicted affinity (median: 81 nM) than those unique to TAPBPR^KD^ cells (median: 132 nM) (**Figure 3G**). This improvement in affinity was recapitulated when comparing the predicted affinities of peptides enriched on TAPBPR^WT^ (median: 77 nM) and TAPBPR^KD^ cells (median: 129 nM) (**Figure 3H**). These data indicate that TAPBPR promotes the presentation of high-affinity peptides on HLA-B*44:02, as was also observed for HLA-B*44:05.

### 3.7 TAPBPR functions as a peptide editor on HLA-B*44:05, loading peptides containing a C-terminal tryptophan residue, but it is an inefficient editor on HLA-B*44:02

**Figure 4:**
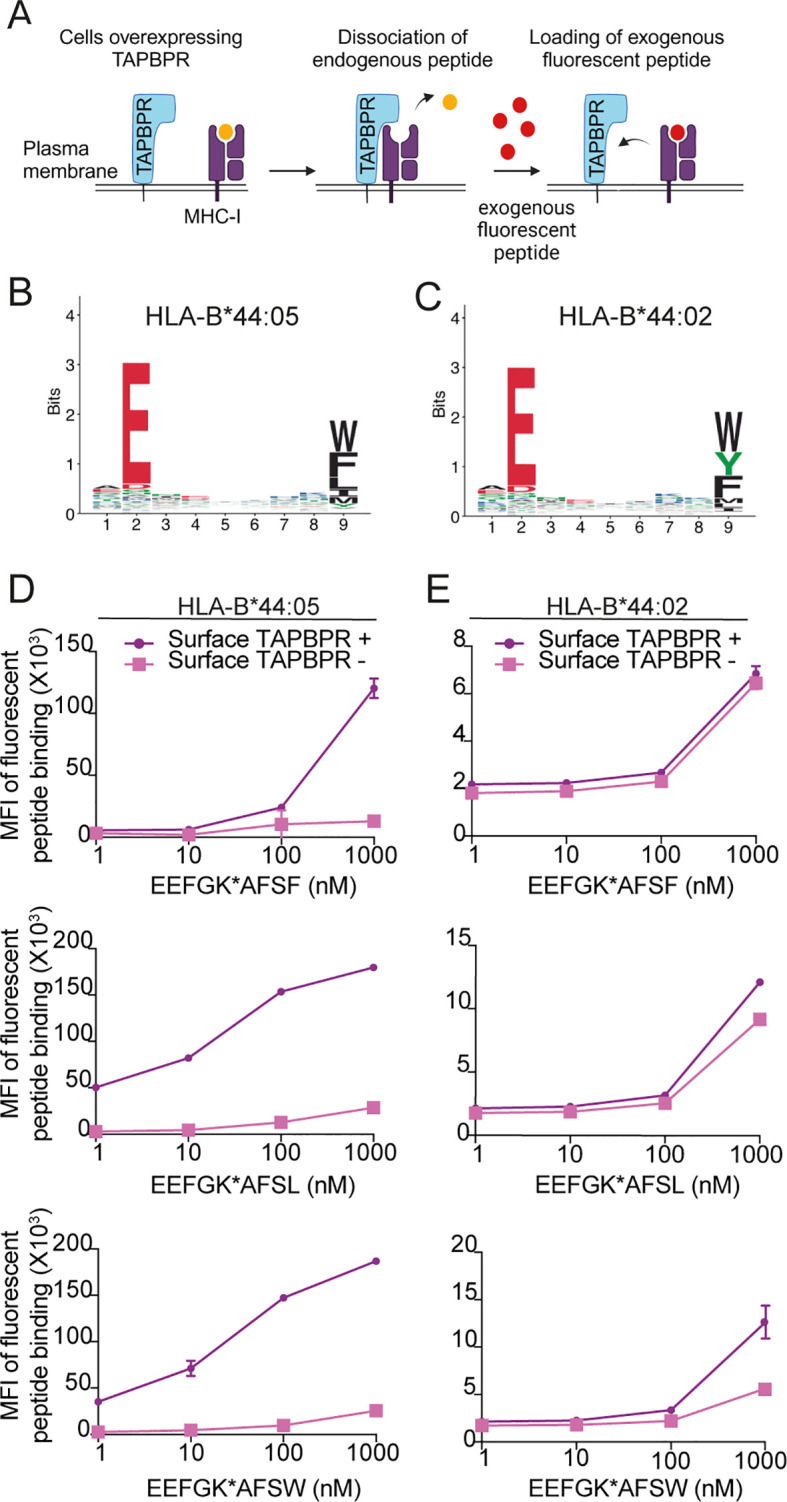
TAPBPR exhibits preferential loading of peptides with C-terminal leucine or tryptophan onto HLA-B*44:05 but is inefficient at loading peptides onto HLA-B*44:02. **(A)** Schematic overview of TAPBPR-mediated peptide loading assay using overexpressed TAPBPR. **(B, C)** Consensus sequence for HLA-presented peptides predicted to bind to **(B)** HLA-B*44:05 and **(C)** HLA-B*44:02 from MHC Class I (mhcmotifatlas.org). **(D)** HLA-B*44:05 or **(E)** HLA-B*44:02 positive cells overexpressing TAPBPR were pre-stimulated with IFNγ and then incubated with fluorescent 9mer peptides EEFGK*AFSF, EEFGK*AFSL, or EEFGK*AFSW (where K* indicates TAMRA-labelled lysine residues) for 1 **(h)** Following harvesting, cells were stained for surface TAPBPR (see **Supplementary Figure 4**), and peptide loading onto surface TAPBPR-positive (purple line) and surface TAPBPR-negative (pink line) was assessed. Bars show MFI ± SD. Data are representative of at least two independent experiments performed in duplicate. TAPBPR, transporter associated with antigen processing; HLA, human leukocyte antigen; MFI, mean fluorescence intensity.

As our immunopeptidomic results demonstrated that TAPBPR presence promoted the presentation of a tryptophan at the C-terminus on HLA-B*44 molecules, particularly on HLA-B*44:05, we sought to confirm these results using TAPBPR-mediated peptide loading assays. Previously, we have shown that the overexpression of TAPBPR in HeLaM cells results in the leakage of TAPBPR: MHC-I complexes to the plasma membrane, which can be utilized to test the ability of TAPBPR to load fluorescently labelled peptides onto HLA-I molecules by flow cytometry (38) (**Figure 4A**). Thus, we generated TAPBPR-overexpressing variants of the HLA-B*44:02 and HLA-B*44:05-expressing cell lines (**Supplementary Figure 4A**), and we confirmed that a small proportion of the cells expressed TAPBPR at the cell surface (**Supplementary Figure 4B-C**, purple line).

We then tested the ability of TAPBPR to promote the loading of three fluorescent 9mer peptides, which vary at their C-terminal residue—EEFGK*AFSF, EEFGK*AFSL, or EEFGK*AFSW—where K* indicates a TAMRA-labelled lysine residue. Based on the consensus sequence of HLA-B*44:05 and HLA-B*44:02 binding peptides (**Figures 4B,C**), EEFGK*AFSF and EEFGK*AFSW were predicted to be able to bind to both HLA-B44 molecules, while EEFGK*AFSL was expected to be HLA-B*44:05 specific. TAPBPR loaded all three peptides onto HLA-B*44:05 (**Figure 4D**). The efficiency of TAPBPR-mediated peptide loading on HLA-B*44:05 was higher for the peptide containing the C-terminal leucine and tryptophan than for the peptide variant containing a phenylalanine at its C-terminus (**Figure 4D**).

In contrast, TAPBPR was generally inefficient at promoting peptide loading onto HLA-B*44:02 (**Figure 4E**). It was unable to load the peptide containing a C-terminal phenylalanine, exhibited minimal loading of the peptide with the C-terminal leucine, and was able to promote some loading of the peptide with the tryptophan at its C-terminus, when high concentrations of peptide (1 µM) were used (**Figure 4E**). Together, these results suggest that HLA-B*44:05 and HLA-B*44:02 differ in their intrinsic ability to undergo TAPBPR-mediated peptide editing, with HLA-B*44:05 being receptive to peptide editing by TAPBPR while HLA-B*44:02 is relatively resistant. One possible reason for this is that peptides presented by HLA-B*44:02 appear to have a higher affinity (median: 72 nM) than those presented by HLA-B*44:05 (median: >600 nM) (**Supplementary Figure 5**), and thus, TAPBPR may not be able to dissociate peptides from HLA-B*44:02 efficiently. Finally, our data suggest that peptides with a C-terminal tryptophan are most efficient at competing with TAPBPR for HLA-B*44 molecules, followed by leucine, while phenylalanine is least efficient.

## 4 Discussion

Here, we explored whether the tapasin dependency of HLA-B*44:02 was due to its inability to interact with TAPBPR, while the tapasin independence of HLA-B*44:05 resulted from its ability to obtain peptide via TAPBPR-mediated peptide exchange. We showed that the interactions of both HLA-B*44:02 and HLA-B*44:05 with TAPBPR are very weak or transient in nature, in line with previous findings for HLA-B molecules (26). In addition to confirming the different tapasin dependence profiles of HLA-B*44:02 and HLA-B*44:05 (21, 24) in HeLaM cells, we showed that TAPBPR loss does not significantly impact the surface expression of either overexpressed HLA-B44 allotype. Thus, neither HLA-B*44:02 nor HLA-B*44:05 is dependent on TAPBPR for surface expression. Furthermore, we showed that TAPBPR shapes the immunopeptidome of both HLA-B*44:02 and HLA-B*44:05 and promotes the loading of high-affinity peptides onto both allotypes. This supports the concept that the role of TAPBPR is to optimize the peptide repertoire (6, 16). However, our findings suggest that the difference in tapasin dependency of the two HLA-B44 allotypes is not based on their intrinsic ability to interact with and be edited by TAPBPR but involves a more complex interplay.

Strikingly, TAPBPR had opposing effects on the size of the peptide repertoire presented by HLA-B*44:02 and HLA-B*44:05. For tapasin-independent HLA-B*44:05, TAPBPR restricted the overall peptide repertoire presented (**Figure 2**). This is in keeping with our previous studies on other HLA allotypes (6, 17). However, for tapasin-dependent HLA-B*44:02, TAPBPR surprisingly broadened the peptide repertoire presented (**Figure 3**). Somewhat contradictory to this are our findings that HLA-B*44:02 is relatively resistant to TAPBPR-mediated exchange while HLA-B*44:05 is receptive (**Figure 4**).

**Figure 5:**
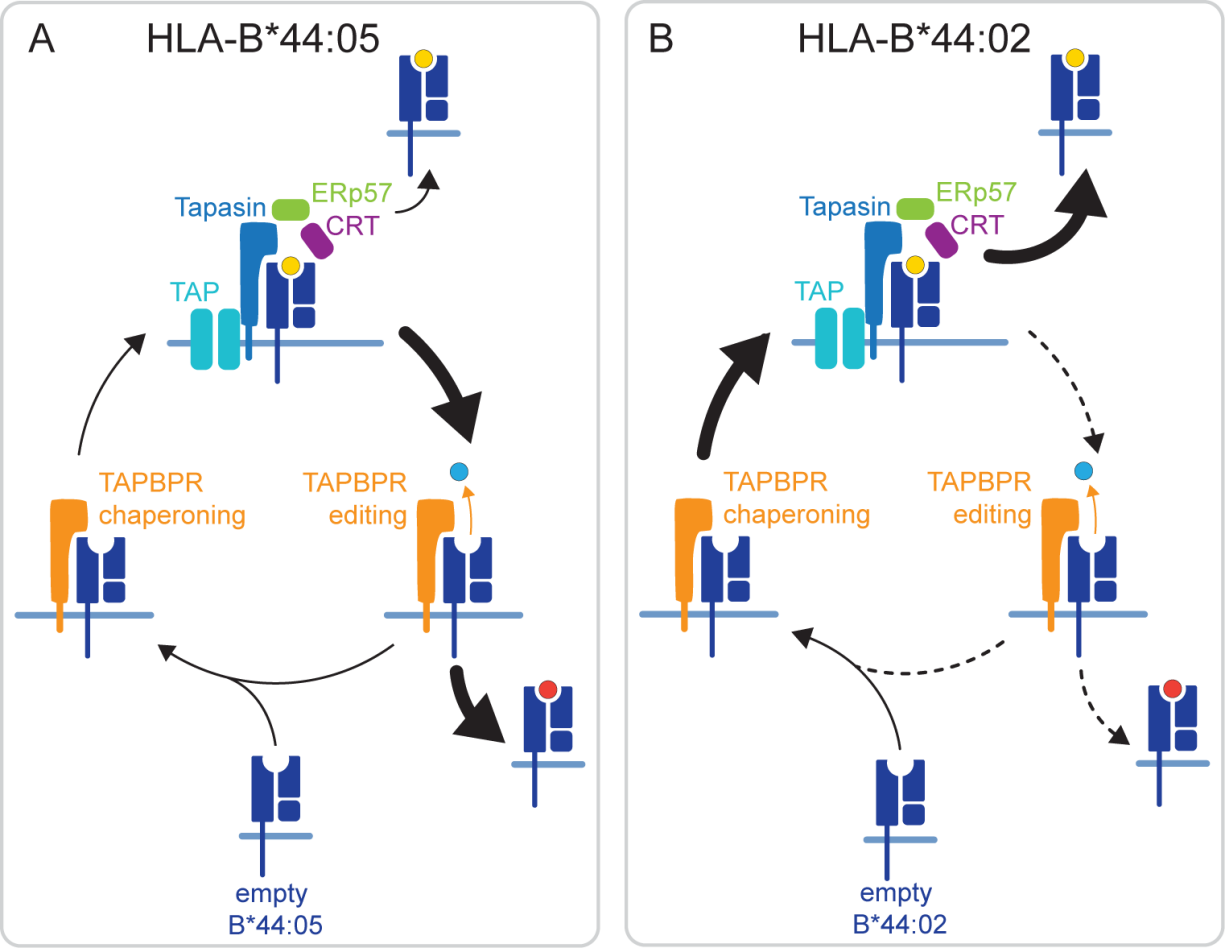
Hypothesized mechanisms by which TAPBPR differentially shapes the immunopeptide displayed in HLA-B*44:05 and HLA-B*44:02. **(A)** HLA-B*44:05 is known for its ability to obtain peptides in a tapasin-independent manner. This will result in a population of HLA-B*44:05 molecules in the ER loaded with suboptimal peptide. We propose that TAPBPR plays a significant role in the removal of these suboptimal peptides from HLA-B*44:05 through its actions as an MHC-I peptide exchange catalyst. This fits with the overall restriction observed in the HLA-B*44:05 peptide repertoire in the presence of TAPBPR and the ability of TAPBPR to perform peptide exchange on this MHC-I molecule. **(B)** In contrast, HLA-B*44:02 is known for being highly dependent on tapasin for peptide loading. Our findings suggest that TAPBPR is inefficient at loading peptides on HLA-B*44:02, but helps broaden the peptide repertoire displayed on this MHC-I molecule. We propose that for HLA-B*44:02, TAPBPR predominantly functions as a chaperone, stabilizing HLA-B*44:02 in a peptide-receptive state, which is subsequently loaded by tapasin in the peptide-rich environment of the PLC. ER, endoplasmic reticulum; TAPBPR, transporter associated with antigen processing; PLC, peptide-loading complex; MHC-I, major histocompatibility complex class I.

The interaction of HLA-B*44:05 with intracellular TAPBPR was particularly difficult to detect, both here and in our previous work (26). This could be due to HLA-B*44:05 being highly stable in a peptide-receptive state, which subsequently results in its ability to bind to peptide in a tapasin-independent manner (23–25). As these spontaneously loaded peptides have not undergone peptide editing, presumably many will be of suboptimal affinity. Indeed, analysis of the predicted affinity of the total peptide pool eluted in our studies confirms that HLA-B*44:05 presents peptides of a wide range of predicted affinity (**Supplementary Figure 5**). Given that HLA-B*44:05 expression only drops by 50% in both peptide editors’ absence, it can obtain a broad peptide repertoire due to its intrinsic properties. We propose that suboptimal p:HLA-B*44:05 complexes undergo TAPBPR-mediated peptide exchange, removing lower-affinity peptides (**Figure 5A**). Subsequently, peptides are replaced quickly with higher-affinity ones on HLA-B*44:05, either directly loaded by TAPBPR or via TAPBPR working with UDP-Glucose : Glycoprotein Glucosyltransferase 1 to promote the incorporation of MHC-I into the peptide-loading complex (39) (**Figure 5A**). This model is consistent with intracellular TAPBPR binding transiently to HLA-B*44:05, recombinant TAPBPR being an effective peptide editor on HLA-B*44:05, the overall restrictive effect of TAPBPR on the HLA-B*44:05 immunopeptidome, and the significant improvement in the affinity of peptides presented in the presence of TAPBPR.

A different mechanism likely underpins the broadening effect of TAPBPR on the immunopeptidome of HLA-B*44:02. Fundamental to the tapasin-dependency of HLA-B*44:02 is its instability in a peptide-unoccupied state (24). In addition to functioning as a peptide exchange catalyst, TAPBPR can also chaperone MHC-I molecules (16). Thus, we propose that this chaperoning function of TAPBPR plays a more significant role for HLA-B*44:02, helping to maintain molecules in the peptide-receptive state (**Figure 5B**). Rather than assisting in peptide loading directly on HLA-B*44:02, we suggest that TAPBPR promotes the incorporation of the peptide-unoccupied HLA-B*44:02 complexes into the peptide-rich environment of the PLC for loading via tapasin (**Figure 5B**), thus explaining how TAPBPR ultimately broadens the peptide repertoire presented by this MHC-I. This model is consistent with the ability of TAPBPR to broaden the HLA-B*44:02 peptide repertoire while being a poor peptide editor on HLA-B*44:02. Furthermore, it fits with the extreme tapasin dependency of this MHC-I for peptide loading; i.e., if TAPBPR could load peptide on HLA-B*44:02, then this MHC-I molecule would not exhibit such a severe phenotype in the absence of tapasin.

Finally, immunopeptidomic analysis has shown that TAPBPR selectively promotes the loading of peptides containing C-terminal tryptophan onto HLA-B*44:05 (**Figure 2**). A slight increase in the presentation of peptides containing a C-terminal tryptophan on HLA-B*44:02 was also observed in TAPBPR-expressing cells (**Figure 3**). These results are the first indication that TAPBPR may affect the sequence motifs of the peptides presented by MHC-I molecules. The mechanism for this is not yet resolved. Still, we would speculate that tryptophan and phenylalanine residues, having a bulky side chain, require substantial widening of the peptide binding groove to bind into the F-pocket. TAPBPR and tapasin are both known to widen the MHC-I peptide binding groove (8, 10–12) and thus would facilitate the loading of these peptides. In line with this, it has recently been reported that tapasin also promotes the loading of peptides containing C-terminal tryptophan residues on HLA-B*44:05 (40).

Our validation using fluorescent peptide loading assays confirmed the ability of TAPBPR to promote the loading of peptides with a C-terminal tryptophan onto HLA-B*44:05 and HLA-B*44:02, albeit significantly less efficiently onto HLA-B*44:02. In contrast to the immunopeptidomic results for HLA-B*44:05, the peptide loading assays suggested that TAPBPR was more efficient at loading peptides with a C-terminal leucine compared to phenylalanine. The results of the cell surface TAPBPR-mediated peptide loading assay may indicate that peptides with a C-terminal tryptophan residue or leucine may have a higher intrinsic affinity for HLA-B*44:05 than those with a C-terminal phenylalanine. This is in line with the previous observation that the efficiency of TAPBPR-mediated peptide loading is positively correlated to the affinity of the peptide for MHC-I (38).

To conclude, our results show that TAPBPR can substantially impact the peptide repertoire of MHC-I molecules to which it does not bind strongly. Furthermore, we demonstrate a role for TAPBPR in selecting a specific peptide sequence on MHC class I molecules. By exploring the role of TAPBPR in shaping the immunopeptidome presented on two highly related molecules, HLA-B*44:05 and HLA-B*44:02, we shed further light on the relationship between tapasin and TAPBPR in peptide selection onto MHC-I. The relationship between these two editors is more complex than initially anticipated and strongly influenced by HLA polymorphisms.

## Data Availability

The mass spectrometry proteomics data have been deposited to the ProteomeXchange Consortium via the PRIDE (41) partner repository with the dataset identifier PXD063219. The datasets analyzed[ANALYZED] for this study can be found in the [PRIDE database] ([https://www.ebi.ac.uk/pride/archive/projects/PXD063219)]. Please see the “Availability of data” section of Materials and data policies in the Author guidelines for more details.
